# First Investigation of the Physiological Distribution of Legacy and Emerging Perfluoroalkyl Substances in Raw Bovine Milk According to the Component Fraction

**DOI:** 10.3390/foods12132449

**Published:** 2023-06-22

**Authors:** Susanna Draghi, Radmila Pavlovic, Alberto Pellegrini, Marco Fidani, Federica Riva, Gabriele Brecchia, Stella Agradi, Francesco Arioli, Daniele Vigo, Federica Di Cesare, Giulio Curone

**Affiliations:** 1 Department of Veterinary Medicine and Animal Sciences, University of Milan, Via dell’Università 6, 26900 Lodi, Italy; susanna.draghi@unimi.it (S.D.); radmila.pavlovic1@unimi.it (R.P.); federica.riva@unimi.it (F.R.); francesco.arioli@unimi.it (F.A.); daniele.vigo@unimi.it (D.V.); giulio.curone@unimi.it (G.C.); 2 Proteomics and Metabolomics Facility (ProMeFa), IRCCS San Raffaele Scientific Institute, Via Olgettina 60, 20132 Milan, Italy; 3 UNIRELAB Srl, Via Gramsci 70, 20019 Settimo Milanese, Italy; a.pellegrini@unirelab.it (A.P.);

**Keywords:** bovine milk, PFASs, lactation, milk fractions, endocrine disruptors, environmental pollution

## Abstract

**Simple Summary:**

Bovine milk is important for human nutrition, especially for infants. However, per-fluoroalkyl substances (PFASs) are harmful chemicals that can contaminate milk and pose a threat to both humans and animals. This study evaluated how 14 different PFASs were distributed within milk by analyzing the content of whole milk, skim milk, and cream. Samples were taken from 23 cows in Northern Italy that were not exposed to known sources of PFASs. The most common PFAS found in all three fractions was perfluorobutanoic acid (PFBA), followed by perfluorooctane sulfonate (PFOS) and perfluorooctanoic acid (PFOA). PFOS was found in higher concentrations in cream compared to raw and skimmed milk. Multiparous cows had higher levels of PFASs in their milk. To fully understand the risks of consuming dairy products contaminated with PFASs and their potential impact on cattle health status, further investigation is necessary. Additionally, more studies are needed to uncover the mechanisms behind the excretion of these substances in milk.

**Abstract:**

Bovine milk is a pillar of the human diet and plays a key role in the nutrition of infants. Perfluoroalkyl substances (PFASs) are well-recognized highly stable organic compounds that are able to pollute ecosystems persistently and threaten both human and animal health. The study aimed to analyze the distribution of 14 PFASs within the milk matrix by comparing their content in whole milk, and its skimmed and creamed fractions. Raw milk samples were individually collected from 23 healthy cows (10 primiparous and 13 multiparous) reared on a farm in Northern Italy not surrounded by known point sources of PFASs. Each sample was fractioned in whole, skim, and cream components to undergo PFAS analysis using liquid chromatography–high-resolution mass spectrometry. All samples contained at least one PFAS, with perfluorobutanoic acid (PFBA) being the primary contaminant in all three fractions, followed by perfluorooctane sulfonate (PFOS) and perfluorooctanoic acid (PFOA). PFOS was shown to be significantly (*p* < 0.001) more concentrated in cream than in raw and skimmed milk. Multiparous cows showed a higher frequency of positive samples in all analyzed fractions. Further research is necessary to assess the risk of dairy diets and high-fat dairy products and to investigate the toxicological effects of PFASs on cattle, even in environments without known PFAS sources.

## 1. Introduction

The mammary gland is responsible for milk production, which represents the only food source for the newborn [1]. For this reason, this secretion provides a great number of different nutrients, such as water, protein, lipids, carbohydrates (mainly lactose), minerals, and vitamins, that are essential for the proper development of the newborn [2,3]. Most of the milk components are produced by the mammary alveolar epithelial cells (AECs), which represent the functional unit of the mammary gland [4]. The AECs recover nutrients from the bloodstream as raw materials and synthesize several milk components (i.e., lactose, caseins, triglycerides), which will be released in the alveolar lumen through the apical membrane [5].

Lactation is recognized as a physiological mechanism that mammals can exploit to increase the clearance of many xenobiotics [6,7]. Due to this close connection with the circulatory stream, the mammary gland contributes to reducing the dam’s body burden by using lactation as a protective additional pathway of toxin excretion [7,8]. Conversely, this mechanism represents a serious threat to the suckling offspring, since milk represents the unique food source for mammalian newborns until the time of weaning [9,10,11]. Newborns are much more susceptible to the adverse effects of toxic substances than adults due to their rapid growth, immature organs, and vulnerable nervous system [12]. In this context, bovine milk plays a key role since it is the most important source of milk worldwide, representing 81% of global production [13], and is a major component of non-breastfed infants’ diets [14]. Several studies have shown that cow’s milk can be a source of different potential toxic substances such as heavy metals, pesticides, mycotoxins, hormones, and other compounds [15,16,17,18,19,20]. Over the last decade, interest has grown in the study of cow’s milk as a possible source of perfluoroalkyl substances, which represent a hot topic in environmental toxicology [21,22]. Several studies investigated the presence of these substances in bovine milk, mainly focusing on different types of processed milk and commercially available dairy products [22,23,24,25], with only one study that explored this topic in Italy [26].

Perfluoroalkyl substances (PFASs) is a nonspecific name that describes a family of more than 9000 synthetic chemicals [27]. In these compounds, the H bound to the carbon chain has been replaced with F atoms. The two most important PFAS subclasses are the perfluorinated sulfonic acids (PFSAs) and carboxylic acids (PFCAs) [28]. In general, these chemicals differ from each other in the number of functional groups and carbon chain length [29,30]. These substances have different physical, chemical, and biological properties, which have led to numerous industrial and commercial applications. The most important properties that have made them so widely used are hydrophobicity and oleophobicity [30]. For example, they are used in paints, polishes, cleaning products, herbicides and insecticides, food packaging, textiles, etc. [30,31,32,33,34]. Indeed, due to their wide use, persistence and mobility, PFASs are largely found everywhere: in soil, surface water and groundwater [35]. Several studies have demonstrated that, thanks to the strength of the carbon-fluorine bond, they resist biodegradation, photooxidation and hydrolysis and, furthermore, exhibit high resistance to metabolic processes and microbial degradation [36]. Moreover, PFASs showed stability in the presence of acids, bases, oxidants and reductants [37]. Due to their environmental persistence, bioaccumulation, and toxicity to wildlife and humans, the production and use of two legacy PFASs, perfluorooctanoic acid (PFOA) and perfluorooctanesulfonic acid (PFOS), have been strongly reduced by the major global manufacturers in the last two decades [27,38] according to the most recent and important regulatory interventions [39]. Many efforts have been made to understand the toxic effects of these substances, and thanks to numerous epidemiological studies, the associations between exposure to specific PFASs and a variety of health effects in humans have been proved. In particular, these substances are able to interfere with the immune system and thyroid function [40], with lipid and insulin regulation [41,42], and they are able to produce adverse reproductive and developmental outcomes [43,44]. Moreover, PFOA and PFOS exposure has also been associated with liver and kidney disease and cancer [45,46,47,48]. Due to their recognized immunotoxicity, in 2020, the European Food Safety Authority (EFSA) established a group tolerable weekly intake (TWI) of 4.4 nanograms per kilogram of body weight per week for the sum of the main perfluoroalkyl substances that accumulate in the body: PFOA, PFOS, perfluorononanoic acid (PFNA), and perfluorohexane sulfonic acid (PFHxS) [49].

Given their recognized ubiquity, the main hypotheses of this work are that PFASs are present in bovine milk from animals reared in an agricultural area in Italy not surrounded by known point sources of contamination and that these substances are physiologically distributed and accumulated in different milk fractions according to their chemical peculiarities. Accordingly, the objective of this study was to analyze the distribution of 14 perfluoroalkyl substances within the milk matrix by comparing their content in whole milk, and its skimmed and creamed fractions, to understand whether a difference in the chemical characteristics of perfluoroalkyl substances could result in a different accumulation pattern within the specific portion of milk.

## 2. Materials and Methods

### 2.1. Chemicals and Reagents

All solvents, 25% ammonia solution, methanol for HPLC LC-MS grade and acetonitrile were purchased by VWR International S.r.l. (Radnor, PA, USA). The ISOmix (ISO21675:2019 native stock solution) containing nine PFCAs (perfluorobutanoic acid (PFBA), perfluoropentanoic acid (PFPeA), perfluorohexanoic acid (PFHxA), perfluoroheptanoic acid (PFHpA), PFOA, PFNA, perfluorodecanoic acid (PFDA), 2H-Perfluoro-2-decenoic Acid (FOUEA) and sodium dodecafluoro-3H-4,8-dioxanonanoate (NaDONA)) and five PFSAs (perfluorobutane sulfonate (PFBS), PFHxS, PFOS, N-methylperfluoro-1-octanesulfonamidoacetic acid (N-MeFOSAA), and sodium 1H,1H,2H,2H-perfluorooctanesulfonate (6:2) (6:2FTS)), ammonium formate, and the two C-labeled internal standards (ISs) perfluoro-[1,2,3,4,5 13C5] nonanoic acid (MPFNA) and perfluoro-[1,2,3,4-13C4] octanesulfonic acid (MPFOS) were purchased from Chemical Research 2000 Srl (Rome, Italy). Water was purified by a Milli-Q system (Millipore, Merck KGaA, Darmstadt, Germany). Individual stock standard solutions of ISs were diluted in MeOH, mixed at a final concentration of 1 ppm and stored at −20 °C.

### 2.2. Sample Collection and Preparation for the Extraction

The study included the milk of 23 healthy cows, 10 primiparous and 13 multiparous (Table 1), reared in Lombardy on a farm that employed standard Italian farming practices and was selected to represent a background contaminated agricultural area not surrounded by known point sources of PFASs. From each animal, an aliquot of 250 mL of raw milk was collected during the morning milking, then samples were transferred under refrigeration conditions at 4 °C to undergo analysis on the same day. Upon arrival at the laboratory, raw milk preparation for extraction involved the consecutive steps described as follows: (1) for each sample, 5 g of whole milk was weighed into a 50 mL tube for PFASs extraction while another 100 g was initially centrifuged at 4000× *g* for 10 min at 4 °C to obtain the separation of cream and skimmed fraction; (2) the resulting cream amount was weighed and transferred into a separate tube, and its weight was registered to later adjust the internal standard (IS); (3) for the skim fraction, 5 g was transferred into another separate tube. All the analyses were performed in duplicate.

### 2.3. Extraction Procedure

Regardless of the milk fraction, all samples were extracted according to a previously validated method [50] with some modifications. After adding the mixture of ISs to achieve a final concentration of 5 ng g^−1^, 10 mL of acetonitrile was added for PFAS extraction and protein precipitation, then samples were homogenized using a high-performance dispersing instrument (T 25 digital ULTRA TURRAX^®^, IKA^®^-Werke GmBH & Co. KG, Staufen, Germany) for 30 s, vortexed, and sonicated for 15 min. After the sonication, samples were centrifuged (2500× *g*, 4 °C, 10 min), and the supernatant was transferred into a new tube and dried in a rotary vacuum centrifuge at 55 °C. The extract was resuspended in 5 mL of purified Milli-Q water and underwent solid phase extraction (SPE) by using specifically designed cartridges for PFASs analysis (Strata PFAS WAX/GCB, 200 mg/50 mg/6 mL, Phenomenex SRL, Castelmaggiore, Italy), according to manufacturer’s instructions. The eluate was dried in a rotary vacuum centrifuge at 55 °C, then samples were resuspended with 100 μL of MeOH and 100 μL mobile phase (90% water with ammonium formate 20 mM and 10% of MeOH), vortexed for 30 s, and transferred into vials for UPLC-HRMS. To avoid the misinterpretation of analytical results caused by the possible presence of traces of PFASs in the material used for sample extraction and purification, two procedural blanks were prepared without the matrix in each extraction session.

### 2.4. UPLC-HRMS Analysis

The UPLC-HRMS system consisted of a Vanquish (Thermo Fisher Scientific, Waltham, MA, USA) (equipped with a binary pump, auto-sampler and thermostat compartment for two columns) coupled to a Thermo Q Exactive OrbitrapTM (Thermo Fisher Scientific, Waltham, MA, USA), equipped with a heated electrospray ionization source. All PFASs were chromatographically separated, slightly modifying a validated method [41] by using a Raptor ARC-18 5 um EXP guard column (Restek, Bellefonte, PA, USA). Moreover, a CMB WR C18 50 × 4.6 mm, 10 μm (PerkinElmer Italia SPA, Milan, Italy), was introduced before the injector to allow delaying of eventual PFASs already present in the system. The mobile phase consisted of phase A (20 mM aqueous ammonium formate) and B (MeOH). The gradient started with 20% B, which reached 95% B at the 20th min and was kept in this condition for 10 min. At the 30th minute, the initial conditions (20% B) were reached and kept for 4 min for requilibration. The run was performed at 0.3 mL min^−1^, with a total duration of 35 min. Regarding the detector parameters, the capillary and vaporizer temperatures were set at 330 and 280 °C, respectively, the sheath and auxiliary gas were set at 35 and 15 arbitrary units, and the electrospray voltage was set at 3.50 kV, operating in negative mode. The full scan (FS) acquisition (70,000 FWHM resolution, scan range 200–950 *m/z*, 1E6 of automatic gain control AGC, maximum injection time of 200 ms) was combined with a data-independent acquisition (DIA) mode for the confirmatory response, based on an inclusion list that operated at 35,000 FWHM resolution, 5E4 AGC target, maximum injection time of 100 ms, and isolation window of 2 *m/z*. Software XcaliburTM 4.3 (Thermo Fisher Scientific) was used for the post-run chromatograms and spectra elaboration.

### 2.5. Method Validation

Validation was performed by evaluation of the following parameters: selectivity, the limit of detection (LOD), the limit of quantification (LOQ), and matrix effect. The selectivity of the method was confirmed through the evaluation of the interferences’ peaks present in the blank samples close to the expected PFAS retention times. To avoid the misinterpretation of analytical results caused by the possible presence of traces of PFASs in the material used for sample extraction and purification, eight procedural blanks were prepared without the matrix in each extraction session. Also, quality control assurance (QA/QC) was performed by analyzing the matrix blank samples (*n* = 5) in order to determine the contribution of PFAS in the unfortified matrices and subtract the concentrations in the final calculations, if needed.

The matrix-matched calibration curves (10–100 pg g^−1^) were constructed by spiking blank samples with the appropriate amount of standard mixture. LOD and LOQ limits were calculated according to the following equations: LOD = 3.3 SD/b and LOQ = 10 SD/b, where SD is the standard deviation of the intercept for low concentration levels and b is the slope of the regression line obtained from the principal calibration curve. The matrix effect was calculated by comparing the peak areas of PFASs spiked after the extraction of a blank sample to the peak areas of standards in a solution mixture, expressed as a percentage.

### 2.6. Statistical Analysis

Preliminary statistical evaluation was performed through the Shapiro–Wilk test; the test revealed that data were not normally distributed. Thus, non-parametric statistical evaluation was applied. In particular, Kruskal–Wallis one-way (non-parametric ANOVA) analysis followed by all pairwise multiple comparison processes (Dwass–Steel–Critchlow–Fligner method) were used to check differences between the three datasets (Skim, cream and whole milk). Statistical analyses were performed using jamovi (Version 1.6) software retrieved from https://www.jamovi.org (accessed on 4 April 2023). A *p*-value of 0.05 was set as significant.

## 3. Results

The method demonstrated a high selectivity, without the presence of any interference close to the retention time of the examined PFASs. Furthermore, retention time repeatability, parent mass constancy and highly matched fragmentation patterns were fully accomplished. The matrix effects ranged between 80% and 120%, revealing the good efficacy of extraction and purification protocol (Table S1). Satisfactory LODs and LOQs showed high method sensitivity (Table 2). Table 2 shows PFASs detected in whole, skim and cream milk as well as the median, minimum and maximum concentration, limit of detection (LOD), limit of quantification (LOQ) and detection frequency of samples with a concentration above the LOD.

In general, at least one perfluoroalkyl compound was detected in the milk from 100% of the animals included in this study. For most of the compounds analyzed, the lower concentration was detected in the raw milk. Among the PFCAs investigated, only PFDA and NADONA were not detected in any of the samples, while the main contaminants in all the three matrices analyzed were the PFBA with an average concentration of 317.81 pg/g in skim, 95.37 pg g^−1^ in cream and 255.61 pg g^−1^ in whole milk, followed by PFOA, PFHxA, PFPeA, PFHpA, FOUEA and PFNA. Regarding the five PFSAs analyzed, just PFOS and 6-2 FTS were detected in the three milk fractions, while PFBS and PFHxS were present just in the cream and NmetFOSAA was not detected. PFOS resulted in the most representative perfluorinated sulfonic acids in cream and whole milk, with a concentration, respectively, of 148.40 pg g^−1^ and 21.64 pg g^−1^, while 6-2 FTS was detected as the highest PFSAs in skim milk at a concentration of 14.33 pg g^−1^. Regarding the frequency of detection in skimmed milk fraction, PFBA was found in 78.26% of the samples representing the most frequently detected compound, followed by PFOS (39.13%), then by all other compounds (about 30%), with PFHpA and 6-2 FTS, which presented the lowest frequency of detection at 17.39%. In milk cream, PFOS was detected in all the samples analyzed (100%), while the second and the third most identified compounds were PFBA (56.52%) and PFHpA (43.48%). Finally, in whole milk, PFBA was shown to be the compound with the highest frequency of identification (78.26%), followed by PFOS (43.48%), while all other compounds were detected in less than 30% of the samples. Table S2 presents the concentrations of the 14 PFASs in skimmed, cream and whole milk fraction from each sample analyzed.

Regarding the comparisons of the concentrations of different PFASs between the three different fractions analyzed (skimmed, cream and whole milk), we found a statistically significant difference only in the case of PFOS (Table 3). In particular, PFOS concentrations in milk cream were significantly higher (*p* < 0.001) than those in skimmed and whole milk. A similar trend was also found for PFHxS, whose concentration tended to be higher (*p* < 0.051) in cream than in skimmed and whole milk.

The frequency of detection of the different PFASs analyzed in the milk fractions divided by primiparous and multiparous cows is reported in Table 4. For all the analyzed matrices and compounds, we observed the same trend, namely that multiparous cows presented, in general, a higher percentage of positive samples. Within the group of primiparous animals, the detection frequency did not exceed 20% for the analyzed compounds. The only exception was PFOS in the cream, identified in 100%, regardless of the parity of the cows. In addition, in multiparous animals, PFBA had a high detection frequency result within all the evaluated fractions, specifically in 100% of the skimmed milk samples, 76.92% of the cream samples, and 92.30% of the raw milk samples. Table S3 outlines the mean concentrations (±standard deviation) of the different PFASs in primiparous and multiparous cows.

## 4. Discussion

In this study, we attempted to assess the concentration of 14 different PFASs, not focusing solely on whole milk, but also analyzing how these substances can distribute within the milk matrix and change their level when we isolate the cream and skimmed fractions. To the best of our knowledge, this represents the first study of this kind. In addition, this research represents the first field exploration in Italy of a dairy farm representing a background contaminated agricultural area not surrounded by known point sources of PFASs that employed milk as a noninvasive biological matrix to investigate animals’ exposure to these substances.

### 4.1. Detection Frequency and Quantification of PFASs in Bovine Milk

Other studies have examined different types of processed milk, but all the works dealt with commercial dairy products, thus with samples not obtained from the same farm and from individual animals. In addition, commercial products undergo packaging processes that have been found to play an important role in increasing the level of PFAS contamination [51,52]. One of the first studies in this field was performed by Wang et al., who screened commercial milk, milk powder and yogurt samples, reporting PFHpA (68%), PFOA, (68%) and PFNA (46%) as the most frequently found substances, in contrast to PFOS, which was detected in a lower number of samples (24%) [25]. That research reported a median concentration of 26 pg g^−1^ for PFOA, 24 pg g^−1^ for PFOS, 56 pg g^−1^ for PFHpA and 67 pg g^−1^ for PFNA in milk. In our study, lower concentrations were detected with 11.54 pg g^−1^ PFOA and 21,64 pg g^−1^ PFOS and 1.64 pg g^−1^ PFHpA, while PFNA could never be found above the LOD. This different result may be since the milk analyzed by Wang and colleagues was a commercial product and was consequently in contact with packaging that is a well-recognized source of PFAS contamination. Moreover, there could be a different environmental load of PFASs in China compared to Italy. In this regard, an Italian study evaluated the presence of PFOA and PFOS in raw milk and other commercial products such as organic and high-quality milk, skimmed milk and milk cream [26]. They detected PFOA only in organic milk and cream, with a range concentration of 0–32 and 0–27 pg mL^−1^, respectively. Conversely, PFOS was found in all of the sample types, in particular with a concentration range of 0–67 pg mL^−1^ in raw milk, 0–26 pg mL^−1^ in skimmed milk and 0–32 pg mL^−1^ in milk cream. In general, the mean concentration of both the legacy PFASs resulted similarly in this study compared to the findings reported by Barbarossa and colleagues regardless of the fraction, but with much higher maximum values (Table 2). Here, an exception is represented by PFOS in cream of milk, for which markedly higher values were detected, being 148 pg g^−1^, our mean concentration, and varying the range from 3 to 543 pg g^−1^. These different results may depend on the fact that our samples were derived not from bulk storage tanks but from individual animals, and thus lack a dilution effect. Another Chinese study by Xing et al. studied the presence of PFOA and PFOS in raw and retail milk and in yogurt derived from the Xinjiang region [24]. Those authors reported an average PFOS concentration of 2.2 ng L^−1^ and a total absence of PFOA in raw milk, while in retail milk, they found average concentrations of 16.2 ng L^−1^ for PFOA and 24.5 ng L^−1^ for PFOS, which are comparable with those we identified of 11.54 pg mL^−1^ and 21.64 pg mL^−1^, respectively. The different concentrations related to raw milk could likely result from the different types of sampling, with 16 of the 24 samples they analyzed being from individual cows and 8 from bulk storage tanks. In a German study, the presence of several PFASs in different products within the dairy processing chain was evaluated, involving raw milk and different types of commercial milk, cheese, yogurt, and other dairy products (butter, whey, cream, etc.) [23]. Comparing our results to those reported by Still and colleagues in raw milk, it can be seen that their concentrations of PFOA 6.2 pg g^−1^ and especially PFBA 6.5 pg g^−1^ were significantly lower than those we identified, namely 11.54 pg g^−1^ and 255.61 pg g^−1^. The same situation occurs considering the milk cream, where Still and colleagues found average concentrations of 4.8 pg g^−1^ for PFBA, 2.9 pg g^−1^ for PFOA, and 18.9 pg g^−1^ for PFOS, which turn out to be much lower than those found in the present study, namely 95.73 pg g^−1^ PFBA, 31.04 pg g^−1^ PFOA and 148.3 pg g^−1^ for PFOS. Considering Still and colleagues published their paper in 2013, it seems easy to explain our findings. In this study, the higher PFBA concentration may be due to the European restrictions applied to the production and use of long-chain PFASs, especially C8-based perfluoroalkyl chemicals such as PFOS and PFOA, together with the indication of their replacement with alternative, short-chain PFASs such as PFBA [36,53,54]. Although this hypothesis may contrast our concentrations of legacy PFASs (i.e., PFOA and PFOS), it is reasonable that their regional load was even higher in Italy during the same years, consequently remaining still higher to date if compared with the environmental exposure of another country (i.e., Germany). In this regard, the results of Barbarossa et al. (2014), which are in line with ours but were commercial products representing milk pools from different animals and farms, further support the latter hypothesis. In addition, it was already demonstrated that maize-produced feed, which represents one of the pillars of the bovine diet, is susceptible to contamination by PFASs, especially tending to accumulate short-chain PFASs such as PFBA [55]. A recent study published by Lie et al. (2022) analyzed raw milk and cow’s feed samples derived from nine Chinese provinces to investigate PFAS contamination. Those authors indicated PFBA (71.6%), PFOS (71.3%), PFOA (40.8%) and PFPeA (40.8%) as the most frequently found substances in raw milk, while the other PFASs were detected in less than 30% of the samples. These results are discordant with those reported in the present study, where only PFBA in milk was detected with a similar frequency (Table 2). Concerning the mean concentration, Lie et al. also reported higher values of PFOS (0.7 ng g^−1^), PFOA (0.5 ng g^−1^) and PFPeA (0.05 ng g^−1^), while PFBA was detected at the lower concentration of 0.13 ng g^−1^. These differences can be attributed to the fact that China has kept unchanged, indeed enhanced, the production and use of long-chain PFASs after the introduction of restrictions in the United States and Europe [56]. This may also explain the lower PFOA and PFOS milk content in the present study, while the increased use of short-chain PFASs in Europe may underlie the ubiquitous PFBA detection together with the higher concentrations.

### 4.2. Physiological Pattern Distribution of PFASs According to the Different Bovine Milk Fraction

Regarding the percentage of samples with PFAS concentrations above the LOD (Table 2), for most of the PFASs analyzed, there seems to be a common identification pattern, i.e., the highest detection frequency appears to be present in dairy cream. The only exception is related to PFBA, as this compound was identified more in the skimmed fractions and raw milk. This result is interesting because several studies have demonstrated that, in contrast to most other pollutants, PFASs do not tend to accumulate in fat tissues but rather bind to serum albumin and other cytosolic proteins [36,57]. Our results seem to indicate that milk fat tends to concentrate these molecules probably due to a preferential compartmentalization mechanism within this specific fraction. Regarding the concentrations of different PFASs in the three milk fractions, only in the case of PFOS was a significant difference found, as the milk cream presented a higher concentration (*p* < 0.001) in comparison to skimmed and whole milk (Table 3). This result, in our opinion, could depend on the fact that perfluorooctane sulfonate acid has an affinity for the lipid component of milk and thus tends to bind to it, or it could be that this substance is secreted together with fat globules by the mammary gland cell. The latter hypothesis relies on the fact that long-chain PFASs, such as PFOS, have a similar structure of fatty acids, allowing these pollutants to use the same transporters to enter mammary cells and be excreted in milk fat globules [58,59]. Other studies, concerning the physiology of lactation in women, demonstrated that exposure to PFASs, in particular PFOS and PFHxS, leads to the alteration of lipid metabolism in the mammary gland, affecting the composition and size of milk fat globules [60]. Consequently, it is possible that PFASs may become part of the fat globules themselves. Of course, these are hypotheses, and further studies are needed to better characterize the relationship that different PFASs have with different milk components. Regarding the other PFASs, they seem to be ubiquitous in the three matrixes; this result could be since, as it is a pilot study, the sample taken is quite small, and it cannot be excluded that by increasing the numerosity, differences between the matrixes could appear.

### 4.3. Detection Frequency of PFASs in Relation to the Number of Lactations

This is the first study that hypothesized and reported a difference between the presence of PFASs in raw milk and the parity/n° of lactation of cattle, considering their age (Table 2). In humans, different studies demonstrated that primiparous women present higher PFAS milk concentrations than multiparous ones [61,62]. Considering the importance of lactation as an additional route of excretion for these substances besides renal and fecal ways [6,63], all animals included in the study were confirmed to exert this protective physiological mechanism, regardless of parity. In contrast to the reports in women, here, multiparous cows showed higher PFAS concentrations in milk for all the detected compounds. This discrepancy could depend on the fact that the primiparous and multiparous cows considered here presented different average lactation days, 325.1 and 184.6, respectively, allowing primiparous to eliminate more PFASs through the mammary pathway. Confirming this explanation, the length of time a woman breastfeeds an infant is reported to affect the PFAS concentrations in human milk [63]. Moreover, a second aspect to consider is that primiparous cows, unlike primiparous women, have a lower ingestion capacity when compared with multiparous animals [64], which might have reduced their potential for exposure to and accumulation of pollutants. Finally, all animals presented at least one PFAS (of the fourteen compounds investigated) in their milk. This finding confirms that the animals included in this study have been exposed to these substances, suggesting the milk matrix, particularly milk cream, to be extremely useful in indicating lactating cows’ exposure noninvasively.

## 5. Conclusions

In conclusion, PFOS demonstrated preferentially segregative behavior in the fat fraction of bovine milk, although further investigation is needed to define the mechanism behind this phenomenon. Furthermore, perfluoroalkyl substances appear ubiquitous in bovine milk collected from individual animals. Due to a dilution effect in bulk milk, the average consumer may be relatively at risk; however, extensive investigations are needed for consumer groups with predominantly dairy diets (e.g., infants and children) and some specific high-fat dairy products (e.g., butter). Finally, the results of this study indicate that even when reared in an environment with a contamination background with no known sources of PFASs, it is likely that cattle are subjected to continuous exposure to these substances, which can be determined in lactating cows by analyzing the milk fat fraction. Although there are no toxicological end-points for PFASs in bovine species, it is possible to assume that because of their known endocrine-disrupting effects, they can induce metabolic, immunological, and reproductive alterations in this species that can unpredictably impact the health status of herds and their sustainability.

## Figures and Tables

**Table 1:** Average productive data of cows involved in the study.

	Parity (*n*)	Days in Milk (Days)	Milk Yield (Kg)	Fat %	Proteins %	Somatic Cell Count (cells/mL)
**Primiparous**		325.1 ± 105	24.53 ± 4.09	3.4 3± 0.4	3.41 ± 0.23	119,800 ± 78,000
**Multiparous**	2.69	184.6 ± 86	28.48 ± 6.29	3.64 ± 0.58	3.17 ± 0.39	183,769 ± 59,000

**Table 2:** Descriptive statistics of concentration (pg g^−1^) of fourteen PFASs in whole, skim and cream milk.

						Percentile				
	Fractions	Mean ± SD		Median	Min–Max	25th	75th	LOD	LOQ	Samples > LOD
*PFBA*	Skimmed	317.81 ± 785.40		59.00	0–3713.20	0.90	262.00	3.60	11.00	60.56%
	Cream	95.73 ± 137.80		21.10	0–494.50	0.00	141.95			56.52%
	Whole Milk	255.61 ± 551.30		77.40	0–2639.30	32.30	224.70			78.26%
*PFPeA*	Skimmed	3.62 ± 5.60		0.00	0–12.90	0.00	10.30	2.80	8.60	30.43%
	Cream	4.34 ± 6.20		0.00	0–16.90	0.00	10.85			34.78%
	Whole Milk	2.28 ± 4.50		0.00	0–12.30	0.00	0.00			21.74%
*PFHxA*	Skimmed	6.86 ± 11.80		0.00	0–29.00	0.00	12.10	2.80	8.40	26.09%
	Cream	12.42 ± 21.10		0.00	0–64.30	0.00	25.75			30.43%
	Whole Milk	3.94 ± 8.60		0.00	0–29.20	0.00	2.10			26.09%
*PFHpA*	Skimmed	4.01 ± 9.40		0.00	0–43.70	0.00	5.80	2.70	8.10	17.39%
	Cream	4.31 ± 7.40		0.00	0–23.90	0.00	6.65			43.48%
	Whole Milk	1.64 ± 3.30		0.00	0–10.60	0.00	0.65			26.09%
*PFOA*	Skimmed	61.43 ± 207.40		0.00	0–1002.70	0.00	66.00	2.30	6.90	30.43%
	Cream	31.04 ± 52.40		0.00	0–174.40	0.00	58.00			34.78%
	Whole Milk	11.54 ± 27.00		0.00	0–86.30	0.00	3.85			26.09%
*PFNA*	Skimmed	0.43 ± 0.66		0.00	0–1.40	0.00	1.40	2.90	8.80	0%
	Cream	1.00 ± 1.40		0.00	0–4.40	0.00	1.40			13.04%
	Whole Milk	0.55 ± 0.70		0.00	0–1.40	0.00	1.40			0%
*PFDA*	Skimmed	N.D.		N.D.	-	-	-	2.90	8.80	0%
	Cream	N.D.		N.D.	-	-	-			0%
	Whole Milk	N.D.		N.D.	-	-	-			0%
*FOUEA*	Skimmed	2.87 ± 5.10		0.00	0–13.10	0.00	2.50	3.30	10.00	26.09%
	Cream	3.10 ± 5.60		0.00	0–18.40	0.00	5.00			30.43%
	Whole Milk	1.32 ± 3.10		0.00	0–10.20	0.00	0.00			17.39%
*NADONA*	Skimmed	N.D.		-	-	-	-	1.80	5.50	0%
	Cream	N.D.		-	-	-	-			0%
	Whole Milk	N.D.		-	-	-	-			0%
*PFBS*	Skimmed	N.D.		-	-	-	-	2.70	8.10	0%
	Cream	0.23 ± 0.50		0.00	0–1.30	0.00	0.00			0%
	Whole Milk	N.D.		-	-	-	-			0%
*PFHxS*	Skimmed	N.D.		-	-	-	-	2.80	8.40	0%
	Cream	1.74 ± 4.90		0.00	0–20.00	0.00	0.00			13.04%
	Whole Milk	N.D.		-	-	-	-			0%
*PFOS*	Skimmed	8.53 ± 22.50		0.00	0–97.10	0.00	5.20	2.10	6.30	39.13%
	Cream	148.30 ± 152.30		89.60	3.10–543.20	68.30	117.40			100%
	Whole Milk	21.64 ± 56.50		1.00	0–250.60	0.00	9.70			43.48%
*NmetFOSAA*	Skimmed	N.D.		-	-	-	-	2.20	6.80	0%
	Cream	N.D.		-	-	-	-			0%
	Whole Milk	N.D.		-	-	-	-			0%
6-2FTS	Skimmed	14.33 ± 36.00		0.00	0–148.00	0.00	0.00	1.90	5.70	17.39%
	Cream	20.58 ± 56.20		0.00	0–236.10	0.00	0.00			17.39%
	Whole Milk	2.83 ± 13.60		0.00	0–65.00	0.00	0.00			4.35%

N.D. = Not detected.

**Table 3:** Comparisons of PFAS concentrations in relation to the milk fraction (skim, cream and whole milk).

		* p *			* p *
*PFBA*	Skim vs. Cream	0.42	*PFBS*	Skim vs. Cream	0.096
	Skim vs. Whole Milk	0.959		Skim vs. Whole Milk	NaN
	Cream vs. Whole Milk	0.297		Cream vs. Whole Milk	0.096
*PFPeA*	Skim vs. Cream	0.955	*PFHxS*	Skim vs. Cream	0.051
	Skim vs. Whole Milk	0.589		Skim vs. Whole Milk	NaN
	Cream vs. Whole Milk	0.388		Cream vs. Whole Milk	0.051
*PFHxA*	Skim vs. Cream	0.647	*PFOS*	Skim vs. Cream	<0 .001
	Skim vs. Whole Milk	0.961		Skim vs. Whole Milk	0.465
	Cream vs. Whole Milk	0.579		Cream vs. Whole Milk	<0 .001
*PFHpA*	Skim vs. Cream	0.696	*NmetFOSAA*	Skim vs. Cream	NaN
	Skim vs. Whole Milk	0.879		Skim vs. Whole Milk	NaN
	Cream vs. Whole Milk	0.378		Cream vs. Whole Milk	NaN
*PFOA*	Skim vs. Cream	0.857	*6-2FTS*	Skim vs. Cream	0.999
	Skim vs. Whole Milk	0.904		Skim vs. Whole Milk	0.319
	Cream vs. Whole Milk	0.501		Cream vs. Whole Milk	0.319
*PFNA*	Skim vs. Cream	0.475	*NADONA*	Skim vs. Cream	NaN
	Skim vs. Whole Milk	0.814		Skim vs. Whole Milk	NaN
	Cream vs. Whole Milk	0.803		Cream vs. Whole Milk	NaN
*PFDA*	Skim vs. Cream	NaN			
	Skim vs. Whole Milk	NaN			
	Cream vs. Whole Milk	NaN			
*FOUEA*	Skim vs. Cream	0.98			
	Skim vs. Whole Milk	0.593			
	Cream vs. Whole Milk	0.477			

NaN = Not analyzed.

**Table 4:** Detection frequency in primiparous and multiparous cows of the different PFASs with a concentration above the limit of detection.

		Skim	Cream	Whole			Skim	Cream	Whole
*PFBA*	Primiparous	40%	20%	60%	*PFBS*	Primiparous	N.D	0%	N.D
	Multiparous	100%	76.92%	92.30%		Multiparous	N.D	0%	N.D
*PFPeA*	Primiparous	10%	10%	10%	*PFHxS*	Primiparous	N.D	10%	N.D
	Multiparous	46.15%	53.84%	30.77%		Multiparous	N.D	15.38%	N.D
*PFHxA*	Primiparous	10%	10%	10%	*PFOS*	Primiparous	10%	100%	10%
	Multiparous	38.46%	46.15%	38.46%		Multiparous	53.85%	100%	69.23%
*PFHpA*	Primiparous	20%	10%	10%	*NmetFOSAA*	Primiparous	N.D	N.D	N.D
	Multiparous	38.46%	46.15%	30.77%		Multiparous	N.D	N.D	N.D
*PFOA*	Primiparous	20%	10%	10%	*6-2FTS*	Primiparous	10%	20%	N.D
	Multiparous	38.46%	53.84%	38.46%		Multiparous	30%	15.38%	7.69%
*PFNA*	Primiparous	0%	0%	0%	*NADONA*	Primiparous	N.D	N.D	N.D
	Multiparous	0%	23.08%	0%		Multiparous	N.D	N.D	N.D
*PFDA*	Primiparous	N.D	N.D	N.D					
	Multiparous	N.D	N.D	N.D					
*FOUEA*	Primiparous	10%	10%	10%					
	Multiparous	38.46%	46.15%	23.08%					

## Data Availability

Data is contained within the article or Supplementary Material.

## Supplementary Materials

The following supporting information can be downloaded at: https://www.mdpi.com/article/10.3390/foods12132449/s1, Table S1: Validation parameters; Table S2: PFASs concentration in bovine milk according to the fraction; Table S3: PFASs concentration primiparous vs. multiparous.
