# Supplementary material for: First Investigation of the Physiological Distribution of Legacy and Emerging Perfluoroalkyl Substances in Raw Bovine Milk According to the Component Fraction
Source: Foods. 2023 Jun 22;12(13):2449. doi: 10.3390/foods12132449 (PMC10340448; doi:10.3390/foods12132449)
Supplement: Supplementary file 1 [file foods-12-02449-s001.zip › foods-2353688-SI.pdf]

Table S1. Validation parameters.

Compound	Formula	Parent Exact Mass m/z	RT (min)	LOD (pg/g)	LOQ (pg/g)	Linearity	R^2	Recovery (%)	Matrix effect (%)	CV Intra-day (%)	CV Inter-day (%)
**PFBA**	C4HF7O2	212.9792	3.71	3.60	11.00	y=0.0095x – 0.0001	0.998	110	82	12	18
**PFPeA**	C5HF9O	262.9760	9.64	2.80	8.60	y=0.0451x – 0.0004	0.990	97	84	10	14
**PFBS**	C4HF9O3S	298.9430	10.90	2.70	8.10	y=0.18x + 0.0010	0.995	114	92	10	16
**PFHxA**	C6HF11O2	312.9728	13.37	2.80	8.40	y=0.0711x + 0.0003	0.999	102	102	14	17
**PFHpA**	C7HF13O2	362.9696	15.53	2.70	8.10	y=0.1118x + 0.00006	0.999	104	112	10	18
**NaDONA**	C7HF12NaO4	375.9683	15.75	1.80	5.50	y=0.2338x + 0.0009	0.998	110	117	12	20
**PFHxS**	C6HF13O3S	398.9366	15.67	2.80	8.40	y=0.184x + 0.0011	0.996	108	120	10	20
**PFOA**	C8HF15O2	412.9664	17.05	2.30	6.90	y=0.1707x + 0.0007	0.990	92	88	9	14
**6:2FTS**	C8H5F13O3S	427.9752	16.96	1.90	5.70	y=0.2563x + 0.0028	0.990	102	82	11	15
**FOUEA**	C10H2F16O	440.9850	18.73	3.30	10.00	y=0.0409x + 0.0003	0.993	92	102	11	17
**PFNA**	C9HF17O2	462.9632	18.27	2.90	8.80	y=0.0907x + 0.0003	0.999	90	98	14	18
**PFOS**	C8HF17O3S	498.9302	18.24	2.10	6.30	y=0.1262x + 0.0006	0.998	94	93	10	18
**PFDA**	C10HF19O2	512.9600	21.92	2.90	8.80	y=0.1987x + 0.0022	0.976	110	108	10	18
**N-MetFOSAA**	C11H6F17NO4S	569.9746	19.91	2.20	6.80	y=0.0448x – 0.0007	0.993	101	87	10	20

Table S2. Concentration (ng g^{-1}) of fourteen PFASs in whole, skim, and cream bovine milk from 23 healthy cows reared in Lombardy on a farm that employed standard Italian farming practices and selected for representing a background contaminated agricultural area not surrounded by known point sources of PFASs.

Milk Fraction	6-2 FTS	PFNA	FOUEA	NADONA	NmetFOSAA	PFBA	PFBS	PFDA	PFHpA	PFHxA	PFHxS	PFOA	PFOS	PFPeA
Skimmed 1	0	0	0	0	0	0,1081	0	0	0	0	0	0	0	0
Skimmed 2	0	0	0	0	0	0,0388	0	0	0	0	0	0	0	0
Skimmed 3	0	0	0	0	0	0,0174	0	0	0	0	0	0	0	0
Skimmed 4	0	0	0	0	0	0,0590	0	0	0	0	0	0	0	0
Skimmed 5	0,1480	0	0	0	0	3,7132	0	0	0,0437	0	0	1,0027	0,0559	0
Skimmed 6	0	0	0	0	0	0	0	0	0	0	0	0	0	0
Skimmed 7	0	0	0	0	0	0	0	0	0	0	0	0	0	0
Skimmed 8	0	0	0	0	0	0,0227	0	0	0	0	0	0	0	0
Skimmed 9	0	0	0	0	0	0,0382	0	0	0	0	0	0	0	0
Skimmed 10	0	0	0	0	0	0	0	0	0	0	0	0	0	0
Skimmed 11	0	0	0	0	0	0	0	0	0	0	0	0	0	0
Skimmed 12	0	0	0	0	0	< LOD	0	0	0	0	0	0	0	0
Skimmed 13	0	0	0	0	0	0	0	0	0	0	0	0	0	0
Skimmed 14	0	0	0	0	0	0	0	0	0	0	0	0	0	0
Skimmed 15	0	< LOD	0,0111	0	0	0,2378	0	0	0,0081	0,0274	0	0,0654	0,0085	0,0118
Skimmed 16	0	< LOD	0,0121	0	0	0,2078	0	0	0,0081	0,0244	0	0,0684	0,0089	0,0127
Skimmed 17	0	< LOD	0,0131	0	0	0,2878	0	0	0,0085	0,0254	0	0,0754	0,0073	0,0128
Skimmed 18	0	< LOD	0,0123	0	0	0,3353	0	0	< LOQ	0,0242	0	0,0675	< LOQ	0,0107
Skimmed 19	0	< LOD	< LOQ	0	0	0,2267	0	0	0,0095	0,0290	0	0,0670	< LOQ	0,0129
Skimmed 20	0	< LOD	0,0123	0	0	0,2862	0	0	0,0100	0,0273	0	0,0666	< LOQ	0,0125
Skimmed 21	0,0716	< LOD	0	0	0	0,3555	0	0	0	0	0	0	0,0971	0,0099
Skimmed 22	0,0652	0	0	0	0	1,2289	0	0	0	0	0	0	0,0082	0
Skimmed 23	0,0447	0	0	0	0	0,1444	0	0	0	0	0	0	< LOD	0
Cream 1	0	< LOD	0	0	0	0,0452	0	0	0	0	< LOD	< LOD	0,4326	0
Cream 2	0	0	0	0	0	0	0	0	0	0	0	0	0,0739	0
Cream 3	0	0	0	0	0	0	0	0	0	0	0	0	0,0985	0
Cream 4	0	0	0	0	0	0,0324	0	0	0	0	0	0	0,1087	0

Cream 5	0	0	0	0	0	0	0	0	0	0	0	0	0,0765	0
Cream 6	0	0	0	0	0	0	0	0	0	0	0	0	0,0705	0
Cream 7	0,2361	0	0	0	0	< LOD	0	0	< LOD	0	0	0	0,0735	0
Cream 8	0	0	0	0	0	0	0	0	0	0	0	0	0,0962	0
Cream 9	0	0	0	0	0	0	0	0	0	0	< LOD	0	0,0896	0
Cream 10	0	0	0	0	0	0	0	0	0	0	0	0	0,0837	0
Cream 11	0	0	0	0	0	0	0	0	0	0	0	0	0,0571	0
Cream 12	0,0357	0	0	0	0	0	< LOD	0	0	0	0,0200	0	0,4345	0
Cream 13	0	0	0	0	0	0	0	0	0	0	0	0	0,0918	0
Cream 14	0	0	0	0	0	0,0211	0	0	0	0	0	0	0,0595	0
Cream 15	0	< LOD	< LOQ	0	0	0,3015	0	0	< LOQ	0,0240	0	0,0709	< LOQ	0,0113
Cream 16	0	< LOQ	0,0113	0	0	0,1733	0	0	0,0114	0,0315	0	0,0822	0,0976	0,0125
Cream 17	0	< LOD	< LOQ	0	0	0,1106	0	0	0,0093	0,0275	0	0,0580	0,0661	0,0123
Cream 18	0	< LOQ	0,0184	0	0	0,3016	0	0	0,0226	0,0643	0	0,1575	0,1261	0,0104
Cream 19	0	< LOD	0,0110	0	0	0,2351	0	0	0,0147	0,0455	0	0,0917	0,0612	0,0140
Cream 20	0	< LOQ	0,0155	0	0	0,4945	< LOD	0	0,0239	0,0639	0	0,1744	0,1524	0,0169
Cream 21	0,0700	< LOD	0	0	0	0,2844	< LOD	0	< LOD	< LOD	0,0130	0	0,4485	0,0101
Cream 22	0,1315	< LOD	0	0	0	0,0897	< LOD	0	< LOD	0	< LOQ	0,0202	0,5432	0
Cream 23	0	< LOD	< LOQ	0	0	0,1106	0	0	0,0093	0,0275	0	0,0580	0,0661	0,0123
Whole 1	0	0	0	0	0	0,0719	0	0	0	0	0	0	0,0068	0
Whole 2	0	0	0	0	0	0,1071	0	0	0	0	0	0	0	0
Whole 3	0	0	0	0	0	0,0419	0	0	0	0	0	0	< LOD	0
Whole 4	0	0	0	0	0	0,1366	0	0	0	0	0	0	< LOD	0
Whole 5	0	0	0	0	0	0,1778	0	0	0	0	0	0	0	0
Whole 6	0	0	0	0	0	0	0	0	0	0	0	0	< LOD	0
Whole 7	0	0	0	0	0	0,0614	0	0	0	0	0	0	0	0
Whole 8	0	0	0	0	0	0,0377	0	0	0	0	0	0	0	0
Whole 9	0	0	0	0	0	0,0270	0	0	0	0	0	0	< LOD	0
Whole 10	0	0	0	0	0	0	0	0	0	0	0	0	0	0
Whole 11	0	0	0	0	0	0	0	0	0	0	0	0	0	0
Whole 12	0	0	0	0	0	0,0704	0	0	0	0	0	0	0	0

Whole 13	0	0	0	0	0	0	0	0	0	0	0	0	0	0
Whole 14	0	0	0	0	0	0	0	0	0	0	0	0	< LOD	0
Whole 15	0	< LOD	< LOQ	0	0	0,3716	0	0	0,0096	0,0192	0	0,0763	0,0142	0,0093
Whole 16	0	< LOD	0	0	0	0,1298	0	0	< LOQ	0,0084	0	0,0122	0,0123	0,0094
Whole 17	0	< LOD	0,0101	0	0	0,2716	0	0	0,0106	0,0292	0	0,0863	0,0182	0,0123
Whole 18	0	< LOD	0,0102	0	0	0,2750	0	0	0,0082	0,0254	0	0,0743	0,0071	0,0119
Whole 19	0	< LOD	0	0	0	0,0774	0	0	< LOD	< LOQ	0	0,0077	< LOQ	0
Whole 20	0	< LOD	< LOQ	0	0	0,1452	0	0	< LOQ	< LOQ	0	0,0087	< LOQ	0,0096
Whole 21	0,0650	< LOD	0	0	0	2,6393	0	0	0	0	0	0	0,2506	0
Whole 22	0	< LOD	0	0	0	0,4657	0	0	0	0	0	0	0,1181	0
Whole 23	0	< LOD	0	0	0	0,7717	0	0	0	0	0	0	0,0592	0

Table S3. Mean concentration (pg g^{-1}) and standard deviation of fourteen PFASs in whole, skim, and cream bovine milk from 23 healthy cows, 10 primiparous and 13 multiparous, reared in Lombardy on a farm that employed standard Italian farming practices and selected for representing a background contaminated agricultural area not surrounded by known point sources of PFASs.

		**Skim (pg g^{-1})**	**Cream (pg g^{-1})**	**Whole (pg g^{-1})**
**PFBA**	Primiparus	401.43±38.78	32.44±94.77	82.6±116.95
	Multiparous	253.48±317.65	144.42±148.93	388.7±709.1
**PFPeA**	Primiparus	1.18±3.73	1.13±3.57	0.93±2.94
	Multiparous	5.50±6.24	6.8±6.76	3.32±5.24
**PFHxA**	Primiparus	2.74±8.66	2.4±7.59	1.92±6.07
	Multiparous	10.02±13.25	20.12±25.05	5.49±10.05
**PFHpA**	Primiparus	5.16±13.77	0.53±1.29	0.96±3.04
	Multiparous	3.05±4.24	7.22±8.79	2.16±3.57
**PFOA**	Primiparus	106.81±315.45	7.09±22.42	7.63±24.13
	Multiparous	26.53±34.99	49.47±61.72	14.55±29.58
**PFNA**	Primiparus	0.14±0.44	0.12±0.32	0.14±0.44
	Multiparous	0.65±0.73	1.66±1.68	0.86±0.71
**PFDA**	Primiparus	0	0	0
	Multiparous	0	0	0
**FOUEA**	Primiparus	1.11±3.51	0.5±1.58	0.5±1.58
	Multiparous	4.22±5.88	5.09±6.71	1.95±3.89
**NADONA**	Primiparus	0	0	0
	Multiparous	0	0	0
**PFBS**	Primiparus	0	0.13±0.41	0
	Multiparous	0	0.3±0.57	0
**PFHxS**	Primiparus	0	2±6.32	0
	Multiparous	0	1.54±3.65	0
**PFOS**	Primiparus	6.44±17.58	102.85±119.15	1.62±4.44
	Multiparous	10.14±26.33	183.26±169.77	37.04±72.47

**NmetFOSAA**	Primiparus	0	0	0
	Multiparous	0	0	0
**6-2FTS**	Primiparus	14.8±46.8	27.18±74.26	0
	Multiparous	13.96±27.14	15.5±39.86	5±18.03
